# Genome-wide identification, structural and gene expression analysis of the bZIP transcription factor family in sweet potato wild relative *Ipomoea trifida*

**DOI:** 10.1186/s12863-019-0743-y

**Published:** 2019-04-25

**Authors:** Zhengmei Yang, Jian Sun, Yao Chen, Panpan Zhu, Lei Zhang, Shaoyuan Wu, Daifu Ma, Qinghe Cao, Zongyun Li, Tao Xu

**Affiliations:** 1 Institute of Integrative Plant Biology, School of Life Sciences, Jiangsu Normal University, Xuzhou, 221116 Jiangsu Province China; 2 Key lab of phylogeny and comparative genomics of the Jiangsu province, Jiangsu Normal University, Xuzhou, 221116 Jiangsu Province China; 3 Department of Plant Biotechnology, College of Agriculture and Life Sciences, Chonnam National University, Gwangju, 500-757 South Korea; 4 Xuzhou Academy of Agricultural Sciences/Sweet Potato Research Institute, CAAS, Xuzhou, 221121 Jiangsu China

**Keywords:** *Ipomoea trifida*, Sweet potato, bZIP transcription factor, Phylogenetic analysis, Gene expression

## Abstract

**Background:**

The basic leucine zipper (bZIP) transcription factor is one of the most abundant and conserved transcription factor families. In addition to being involved in growth and development, bZIP transcription factors also play an important role in plant adaption to abiotic stresses.

**Results:**

A total of 41 *bZIP* genes that encode 66 proteins were identified in *Ipomoea trifida*. They were distributed on 14 chromosomes of *Ipomoea trifida*. Segmental and tandem duplication analysis showed that segmental duplication played an important role in the *ItfbZIP* gene amplification. *ItfbZIPs* were divided into ten groups (A, B, C, D, E, F, G, H, I and S groups) according to their phylogenetic relationships with *Solanum lycopersicum* and *Arabidopsis thaliana*. The regularity of the exon/intron numbers and distributions is consistent with the group classification in evolutionary tree. Prediction of the *cis*-acting elements found that promoter regions of *ItfbZIPs* harbored several stress responsive *cis*-acting elements. Protein three-dimensional structural analysis indicated that ItfbZIP proteins mainly consisted of α-helices and random coils. The gene expression pattern from transcriptome data and qRT-PCR analysis showed that *ItfbZIP* genes expressed with a tissue-specific manner and differently expressed under various abiotic stresses, suggesting that the *ItfbZIPs* were involved in stress response and adaption in *Ipomoea trifida*.

**Conclusions:**

Genome-wide identification, gene structure, phylogeny and expression analysis of *bZIP* gene in *Ipomoea trifida* supplied a solid theoretical foundation for the functional study of *bZIP* gene family and further facilitated the molecular breeding of sweet potato.

**Electronic supplementary material:**

The online version of this article (10.1186/s12863-019-0743-y) contains supplementary material, which is available to authorized users.

## Background

Transcription factors (TFs) are active proteins that recognize and bind to specific sites on a promoter to activate or inhibit gene expression [1, 2]. The basic leucine zipper (bZIP) TFs is one of the most diverse families of TFs [3, 4]. Structurally, this family contains a highly conserved bZIP domain (60–80 amino acids long) which is divided into two parts: the basic region and the leucine zipper region [5]. The basic region, which is the most conserved core part of the bZIP TF, consists of approximately 16 amino acid residues. This region contains an invariant motif N-X7-R/K-X9, which is mainly involved in nuclear localization and target DNA binding function. Meanwhile, the leucine zipper region is less conserved and composed of heptad repeats of Leu or other bulky hydrophobic amino acids (Ile, Val, Phe or Met) positioning exactly the nine amino acids towards the C terminus [1, 2]. Through the interaction of the hydrophobic amino acids in the helical region, the two subunits are tightly bound together to form a coiled-coil dimer structure. This structure affects the binding characteristics, expression diversity and gene regulation of the target gene.

To date, the *bZIP* gene family has been widely identified by genome-wide analyses in various plants, such as 75 *AtbZIPs* identified in *Arabidopsis thaliana* [6], 89 *OsbZIPs* in *Oryza sativa* [3], 131 *GmbZIPs* in *Glycine max* [7], 92 *SbbZIPs* in *Sorghum bicolor* [8], 125 *ZybZIPs* in *Zea mays* [9], 55 *VvbZIPs* in *Vitis vinifera* [10], 64 *CsbZIPs* in *Cucumis sativus* [11], 77 *MebZIPs* in *Maninot esculenta crantz* [12], 112 *MdbZIPs* in *Malus domestica Borkh* [13] and 247 *BrbZIPs* in *Brassica napus* [14]. bZIP TFs are involved in the regulation of seed development [15, 16], cell elongation [17, 18], vascular development [17], flower development [19–22], somatic embryogenesis [23] and nitrogen/carbon and energy metabolism [24–26]. In addition of the essential functions of *bZIPs* in plant growth and development, *bZIPs* play important roles in plants under abiotic stress conditions. *AtbZIP17*, *AtbZIP24*, *OsbZIP12*, *OsbZIP72*, *OsABF1*, *ThbZIP1*, *GmbZIP44*, *GmbZIP62* and *GmbZIP78* directly or indirectly positively regulate the salt stress adaption in plants [7, 27–32]. *OsbZIP52*/*RISBZ5*, *OsbZIP16*, *OsbZIP23*, *OsbZIP45*, *AREB1*, *AREB2*, *ABF3* and *ThbZIP1* are involved in drought tolerance [33–36]. LIP19 is a Fos-like molecular switch in cold signalling [37]. *OsbZIP52*/*RISBZ5* negatively regulates cold stress response [33]. *OsbZIP72* is a positive regulator of ABA response [29], and overexpression of *GmbZIP44*, *GmbZIP62* and *GmbZIP78* attenuates ABA sensitivity [7]. However, the *bZIP* gene family has not been characterized in sweet potato.

Sweet potato (*Ipomoea batatas*) of the family Convolvulaceae is an annual or perennial herb with an extremely high economic value. To date, sweet potato is the seventh largest crop in the world. It is an important food crop, forage crop and a new type of bio-energy crop in China. Sweet potato is a hexaploid cultivar with 90 chromosomes. Due to its large genome and high genetic heterogeneity, the whole genome sequencing and assembly as well as the other related genomics research is very complicated. However, diploid *Ipomoea trifida* G. Don (2n = 2x = 30) which belongs to Batata section B of the genera *Ipomoea*, is considered as an ancestral species of hexaploid cultivated sweet potato [38–40]. The diploid *Ipomoea trifida* is an ideal model species for studying self-incompatibility, genetic mapping, physical mapping, sweet potato breeding, sweet potato transgenic system construction and whole genome sequencing due to its small size, low ploidy, small chromosome number and simple genetic manipulation. In 2017, the genome data of diploid sweet potato variety *Ipomoea trifida* were released (http://sweetpotato.plantbiology.msu. edu/), making it possible to identify and analyse important gene families at the whole genome level in *Ipomoea trifida* [41].

In this study, *bZIP* gene family members were identified in *Ipomoea trifida*. Using various bioinformatics tools, we performed *ItfbZIP* subfamily classification, gene intron/exon distribution analysis, conserved domain and three-dimensional structural homology modeling prediction. And the *ItfbZIP* gene expression profiles generated from RNA-seq were confirmed by quantitative RT-PCR in different plant tissues under various abiotic stresses. This study could be helpful for further functional study of *bZIP* genes and molecular breeding of sweet potato.

## Results

### Identification and designation of bZIP TFs in the *Ipomoea trifida* genome

A total of 66 ItfbZIP proteins encoded by 41 *ItfbZIP* genes were identified. They all include at least one bZIP domain (bZIP_1: PF00170.20, bZIP_2: PF07716.14 or bZIP_Maf: PF03131). The *ItfbZIP* genes were numbered according to its position on the chromosome as *ItfbZIP1~ItfbZIP41*. Different transcripts encoded by the same gene were given the similar names. For instance, 3 transcripts of *ItfbZIP9* gene are ItfbZIP9.1, ItfbZIP9.2 and ItfbZIP9.3 (Additional file 1: Table S1). At the same time, ItfbZIP protein size (aa), MW, pI, subcellular location and phosphorylation site were analyzed (Table 1). The length of ItfbZIP proteins ranges from 158 AA (ItfbZIP41.1) to 754AA (ItfbZIP40), MW varies from 17,227.12 (ItfbZIP41.1) to 81,305.95 (ItfbZIP40) Da and pI distributes from 5.61 (ItfbZIP14) to 9.69 (ItfbZIP41.1). They are all predicted to be located in the nucleus. We used the online tool P3DB to predict the phosphorylation sites of the ItfbZIPs. As listed in Table 1, the ItfbZIPs contain 4 to 28 phosphorylatio sites, wherein the maximum number of phosphorylation sites is 28 for ItfbZIP34.1 and ItfbZIP34.2. The minimum number of phosphorylation sites is 4 for ItfbZIP25.2 and ItfbZIP27.2. About 60% of the ItfbZIPs contain 10 or more phosphorylation sites. Up to 80% of the ItfbZIPs contain more Ser phosphorylation sites than Thr phosphorylation sites.

**Table 1:** Characteristics of *ItfbZIPs* in *I. trifida*

Gene name	Gene ID	Amino acids	MW(Da)	PI	Subcellular location	No. Of phosphorylation site		
						Ser site	Thr site	Total
*ItfbZIP1*	itf01g16700.t1	159	17,242.26	9.69	Nucleus.	3	2	5
*ItfbZIP2*	itf01g24710.t1	468	51,081.9	7.23	Nucleus.	10	4	14
*ItfbZIP3.1*	itf01g27620.t1	432	46,967.07	6.22	Nucleus.	7	7	11
*ItfbZIP3.2*	itf01g27620.t2	317	33,951.71	5.63	Nucleus.	13	7	20
*ItfbZIP4*	itf02g14930.t1	322	35,055.77	5.66	Nucleus.	7	4	11
*ItfbZIP5*	itf03g14110.t1	340	37,151.20	6.2	Nucleus.	9	7	16
*ItfbZIP6*	itf03g16790.t1	292	32,438.48	6.19	Nucleus.	4	1	5
*ItfbZIP7.1*	itf03g29500.t1	372	42,071.81	6.43	Nucleus.	8	5	13
*ItfbZIP7.2*	itf03g29500.t2	324	36,655.73	6.35	Nucleus.	5	4	9
*ItfbZIP8*	itf04g28510.t1	173	20,455.39	6.92	Nucleus.	2	5	7
*ItfbZIP9.1*	itf04g33190.t1	328	36,566.17	7.07	Nucleus.	5	5	10
*ItfbZIP9.2*	itf04g33190.t4	285	31,492.39	5.74	Nucleus.	5	3	8
*ItfbZIP9.3*	itf04g33190.t6	295	32,815.01	9.2	Nucleus.	3	2	5
*ItfbZIP10.1*	itf05g21690.t1	383	43,053.99	6.28	Nucleus.	5	5	10
*ItfbZIP10.2*	itf05g21690.t2	373	42,042.89	6.54	Nucleus.	7	4	11
*ItfbZIP10.3*	itf05g21690.t3	335	37,582.79	6.12	Nucleus.	4	5	9
*ItfbZIP11*	itf05g23650.t1	227	24,148.57	9.62	Nucleus.	4	3	7
*ItfbZIP12*	itf05g12890.t1	375	42,461.33	5.53	Nucleus.	11	2	13
*ItfbZIP13*	itf05g01550.t1	335	37,844.91	5.91	Nucleus.	11	2	13
*ItfbZIP14*	itf05g02250.t1	347	37,706.85	5.61	Nucleus.	12	8	20
*ItfbZIP15.1*	itf06g21610.t1	453	49,470.31	7.82	Nucleus.	7	8	15
*ItfbZIP15.2*	itf06g21610.t2	374	40,435.16	8.51	Nucleus.	6	7	13
*ItfbZIP16*	itf07g01470.t1	327	36,572.2	8.66	Nucleus.	3	5	8
*ItfbZIP17.1*	itf07g00390.t1	482	53,284.76	6.22	Nucleus.	7	6	13
*ItfbZIP17.2*	itf07g00390.t2	501	55,317.93	6.47	Nucleus.	4	3	7
*ItfbZIP17.3*	itf07g00390.t3	414	45,910.63	7.22	Nucleus.	4	2	6
*ItfbZIP17.4*	itf07g00390.t4	404	44,649.24	6.64	Nucleus.	5	3	8
*ItfbZIP18*	itf08g03360.t1	455	49,697.54	6.1	Nucleus.	13	4	17
*ItfbZIP19.1*	itf08g03030.t1	178	20,232.97	10.3	Nucleus.	1	5	6
*ItfbZIP19.2*	itf08g03030.t2	181	20,703.43	10.3	Nucleus.	1	4	5
*ItfbZIP19.3*	itf08g03030.t4	166	18,873.5	10.52	Nucleus.	2	3	5
*ItfbZIP20*	itf09g13580.t1	540	58,567	6.09	Nucleus.	6	5	11
*ItfbZIP21*	itf09g11330.t1	469	51,205.22	7.86	Nucleus.	7	5	12
*ItfbZIP22.1*	itf09g10570.t1	331	36,463.9	6.41	Nucleus.	3	8	11
*ItfbZIP22.2*	itf09g10570.t2	316	34,757.9	6.02	Nucleus.	3	7	10
*ItfbZIP23.1*	itf09g04120.t1	369	41,756.38	8.46	Nucleus.	11	4	15
*ItfbZIP23.2*	itf09g04120.t2	322	36,206.8	9.2	Nucleus.	8	5	13
*ItfbZIP24.1*	itf09g00830.t1	593	64,463.21	6.8	Nucleus.	15	4	19
*ItfbZIP24.2*	itf09g00830.t2	495	53,646.59	6.62	Nucleus.	13	5	18
*ItfbZIP25.1*	itf09g13780.t1	286	31,976.66	5.94	Nucleus.	4	3	7
*ItfbZIP25.2*	itf09g13780.t2	235	26,356.41	6.33	Nucleus.	2	2	4
*ItfbZIP25.3*	itf09g13780.t3	445	49,462.53	6.42	Nucleus.	8	5	13
*ItfbZIP25.4*	itf09g13780.t4	394	43,842.28	6.76	Nucleus.	7	7	14
*ItfbZIP25.5*	itf09g13780.t7	429	47,877.84	7.29	Nucleus.	7	6	13
*ItfbZIP26*	itf09g15920.t1	176	19,677.33	9.21	Nucleus.	3	2	5
*ItfbZIP27.1*	itf09g23000.t1	205	22,960.18	5.49	Nucleus.	5	6	11
*ItfbZIP27.2*	itf09g23000.t2	182	20,462.37	5.79	Nucleus.	2	2	4
*ItfbZIP28.1*	itf09g23040.t1	438	46,873.95	5.62	Nucleus.	9	4	13
*ItfbZIP28.2*	itf09g23040.t2	433	46,272.23	5.52	Nucleus.	12	5	17
*ItfbZIP29*	itf10g21980.t1	359	41,241.48	7.29	Nucleus.	10	3	13
*ItfbZIP30*	itf10g22380.t1	290	30,693.36	8.53	Nucleus.	4	5	9
*ItfbZIP31*	itf10g25610.t1	289	31,158.79	6.19	Nucleus.	3	4	7
*ItfbZIP32*	itf10g09640.t1	436	47,072.45	6.09	Nucleus.	11	6	17
*ItfbZIP33*	itf11g03980.t1	312	34,536.45	5.55	Nucleus.	8	1	9
*ItfbZIP34.1*	itf12g08780.t1	549	56,965.27	6.2	Nucleus.	18	10	28
*ItfbZIP34.2*	itf12g08780.t2	553	57,378.75	6.33	Nucleus.	16	12	28
*ItfbZIP35*	itf12g23960.t1	349	38,224.45	5.75	Nucleus.	12	7	19
*ItfbZIP36*	itf14g02130.t1	479	52,507.02	5.78	Nucleus.	8	4	12
*ItfbZIP37.1*	itf14g09670.t1	328	36,764.3	8.64	Nucleus.	4	5	9
*ItfbZIP37.2*	itf14g09670.t3	294	32,965.99	9.29	Nucleus.	2	3	5
*ItfbZIP38.1*	itf14g12010.t1	512	56,187.72	6.47	Nucleus.	8	4	12
*ItfbZIP38.2*	itf14g12010.t2	508	55,855.41	6.47	Nucleus.	8	5	13
*ItfbZIP39*	itf15g02970.t1	546	60,189.56	6.82	Nucleus.	12	4	16
*ItfbZIP40*	itf15g04720.t1	754	81,305.95	6.25	Nucleus.	13	5	18
*ItfbZIP41.1*	itf15g19800.t1	158	17,227.12	9.69	Nucleus.	3	3	6
*ItfbZIP41.2*	itf15g19800.t2	184	20,395.82	7.03	Nucleus.	3	3	6

### Chromosome localization and duplication of the *ItfbZIP* gene family

**Fig. 1:**
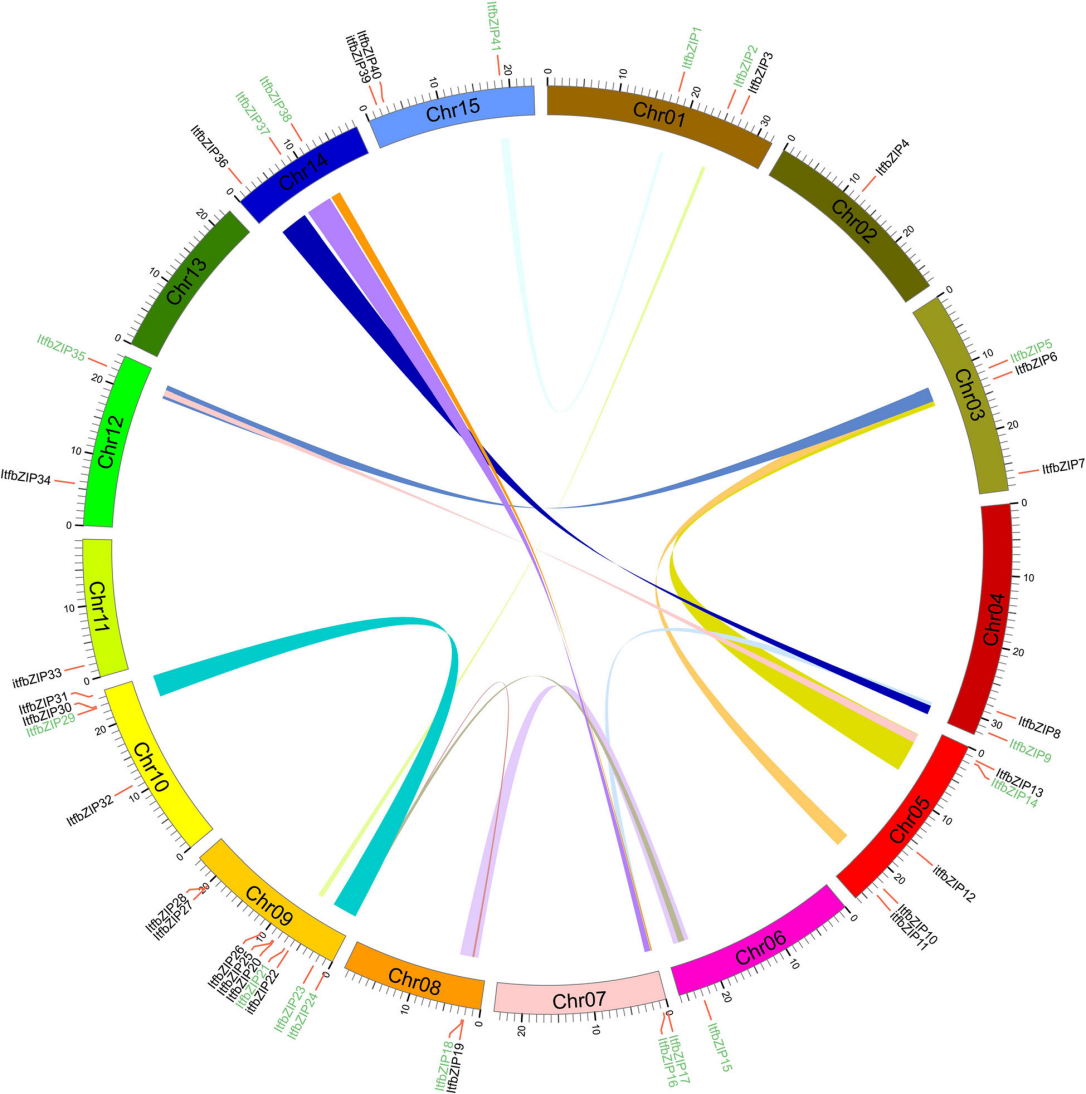
Distribution and segmental duplication of *ItfbZIP* genes in *Ipomoea trifida* Chromosomes. 41 *ItfbZIP* genes were mapped to the 14 chromosomes. Different colored lines indicated the segmental duplication. The red line next to the name indicates the gene cluster on each chromosome. Gene names with collinearity are colored in green, and no collinear gene names are colored in black. The chromosomal location and segmental duplication of the *ItfbZIP* genes are in Table S1

The *ItfbZIP* genes were mapped on 15 chromosomes. As shown in Fig. 1, nine *ItfbZIP* genes on Chr 9; five *ItfbZIP* genes on Chr 5; four on Chr 10; three on Chr 1, Chr 3, Chr 14 and Chr 15; two on Chr 4, Chr 7, Chr 8 and Chr 12; only one on Chr 2, Chr 6 and Chr 11; and no *ItfbZIP* gene on Chr 13, indicating that the distribution of *ItfbZIP* genes is disproportionate on chromosomes.

The amplification of gene family in plant evolution is mainly carried out by genome duplication [42, 43]. To investigate possible relationships among the *ItfbZIP* genes and potential gene duplication type, we performed collinear analysis. (Fig. 1 and Additional file 1: Table S1). According to Holub’s description [44], a chromosomal region within 200 kb containing two or more genes was defined as a tandem duplication event. The results indicated that the *ItfbZIP* gene has no tandem duplication. Segmental duplications were identified using the BLASTP and MCScanX methods and 13 segmental duplications were found. As followers: *ItfbZIP1-ItfbZIP41, ItfbZIP2-ItfbZIP21*, *ItfbZIP5-ItfbZIP14*, *ItfbZIP5-ItfbZIP35*, *ItfbZIP9-ItfbZIP16*, *ItfbZIP9-ItfbZIP37*, *ItfbZIP14-ItfbZIP5*, *ItfbZIP15-ItfbZIP18*, *ItfbZIP15-ItfbZIP24*, *ItfbZIP16-ItfbZIP37*, *ItfbZIP17-ItfbZIP38*, *ItfbZIP18-ItfbZIP24*, *ItfbZIP23-ItfbZIP29.*

### Distribution of bZIP TFs in eukaryotes

**Fig. 2:**
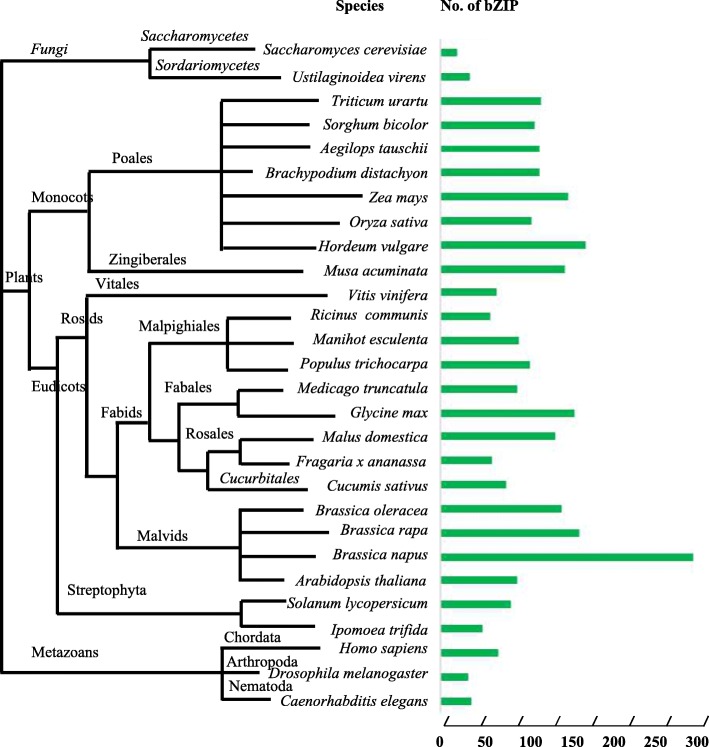
Distribution of bZIP transcription factors in eukaryotes. The total number of *bZIP* genes found in each species is displayed on the right

To understand the evolutionary relationship of bZIP TFs among different eukaryotes, we performed phylogenetic tree construction on 28 species (including fungi, metazoa and plants) and annotated every species with the number of bZIPTFs identified in previous literatures (Fig. 2 and Additional file 2: Table S2). The figure shows only a few bZIP homologs exist in *Saccharomyces cerevisiae* (17) and *Ustilaginoidea virens* (28). However, plants have a large number of *bZIP* genes. The number of bZIPTFs in monocotyledonous plants ranges from 89 to 98 with exceptions of 125 for *Zea mays* and 141 for *Hordeum vulgare,* which may be due to the common ancestor and similar complete genome duplication of the gramineous plants [45]. The range of bZIPTFs in dicotyledonous plants is 41 to 247, wherein the maximum number of bZIPTFs in tetraploid *Brassica napus* is 247, probably due to the large number of gene duplications. Although *Ipomoea trifida* and *Solanum lycopersicum* belong to Solanales, the number of *ItfbZIP* gene (41) is 28 less than that of *SlbZIPs* (69), indicating that the *bZIP* gene number of *Ipomoea trifida* has been reduced during evolution. In general, the numbers of *bZIPs* in *Homo sapiens* and higher plants are more than that in fungus, lower animals and plants, which may be closely related to the ability to regulate physiological responses to environmental stimuli in eukaryotic species.

### Phylogenetic relationship of bZIP proteins in *Arabidopsis thaliana*, *Solanum lycopersicum* and *Ipomoea trifida*

**Fig. 3:**
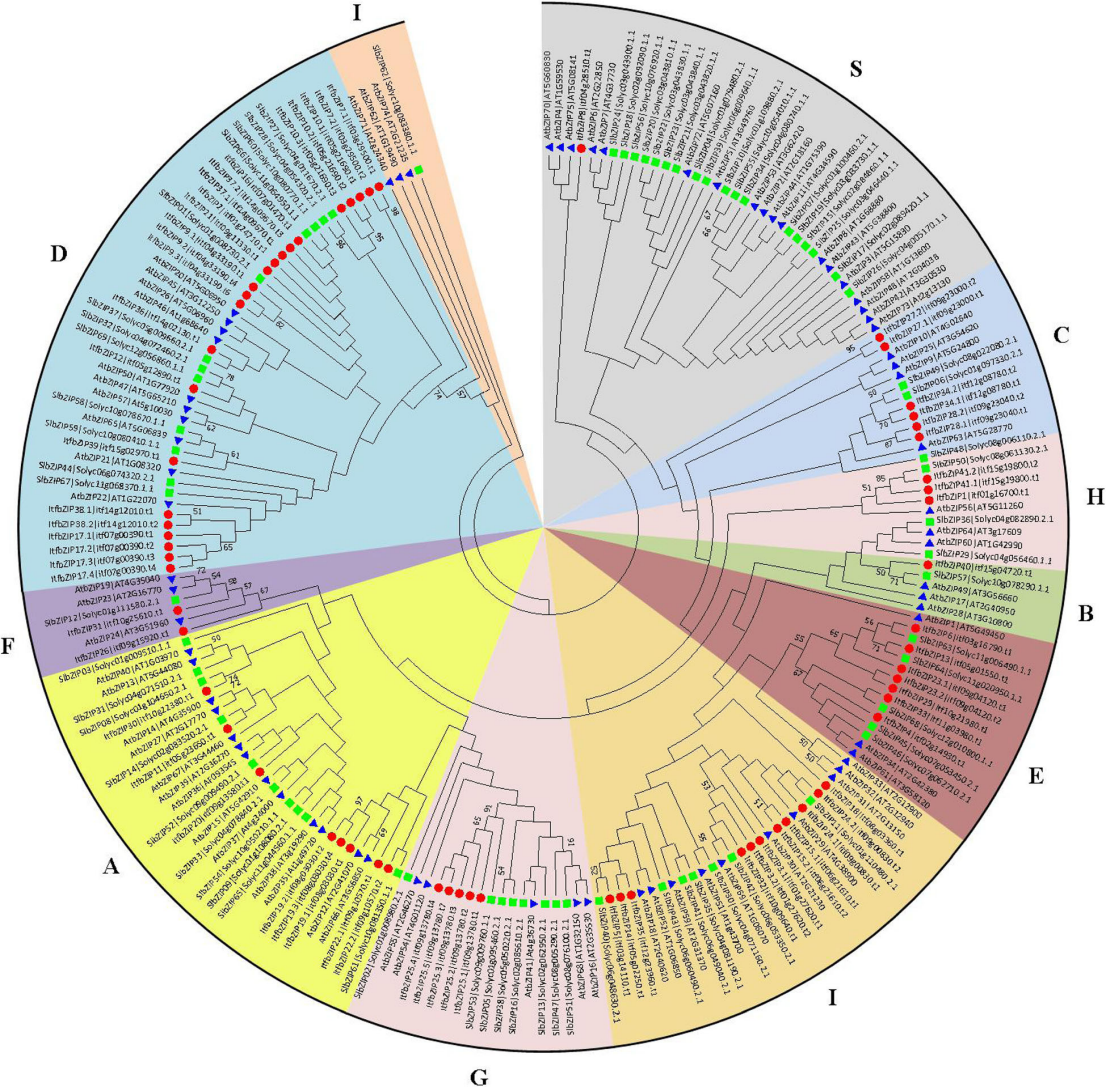
Phylogenetic relationships among the identified bZIP proteins in *Arabidopsis thaliana*, *Solanum lycopersicum* and *Ipomoea trifida*. The 75 *Arabidopsis thaliana*, 69 *Solanum lycopersicum* and 66 *Ipomoea trifida* bZIP domain protein sequences were aligned by ClustalX, and the phylogenetic tree was constructed using MEGA7 by the Maximum Likelihood method analysis (1000 replicates). *Arabidopsis thaliana*, *Solanum lycopersicum* and *Ipomoea trifida* genes are indicated at the end of the branches. Subgroups A–I and S were named according to *Arabidopsis thaliana* [6]. The colored regions indicate different subfamilies, the red solid circles indicate the ItfbZIP proteins, the green solid circles represent the SlbZIP proteins, and the blue solid circles represent the AtbZIP proteins

To study the evolutionary relationship of bZIPs among *Ipomoea trifida*, *Arabidopsis thaliana* and *Solanum lycopersicum*, we established phylogenetic tree of bZIPs of these three species using MEGA7 by Maximum Likelihood method with bootstrap analysis (1000 replicates) (Fig. 3 and Additional file 3: Table S3). The phylogenetic tree consists of 75 *Arabidopsis thaliana*, 69 *Solanum lycopersicum* and 66 *Ipomoea trifida* bZIP protein sequences. The 75 bZIP TFs identified in *Arabidopsis thaliana* were divided into 10 subgroups of A-I and S [6]. The specific distribution of the ItfbZIP proteins was as follows: A (8), B (1), C (6), D (22), E (7), F (2), G(5), H(3), I(11) and S(1). Among the 66 ItfbZIPs, 18% of the ItfbZIPs is close related AtbZIPs, and 72% of the ItfbZIPs is close related SLbZIPs, which is consistent with that *Ipomoea trifida* is belonged to Solanales together with *Solanum lycopersicum* rather than *Arabidopsis thaliana*.

### Gene structure and conserved domain analysis

**Fig. 4:**
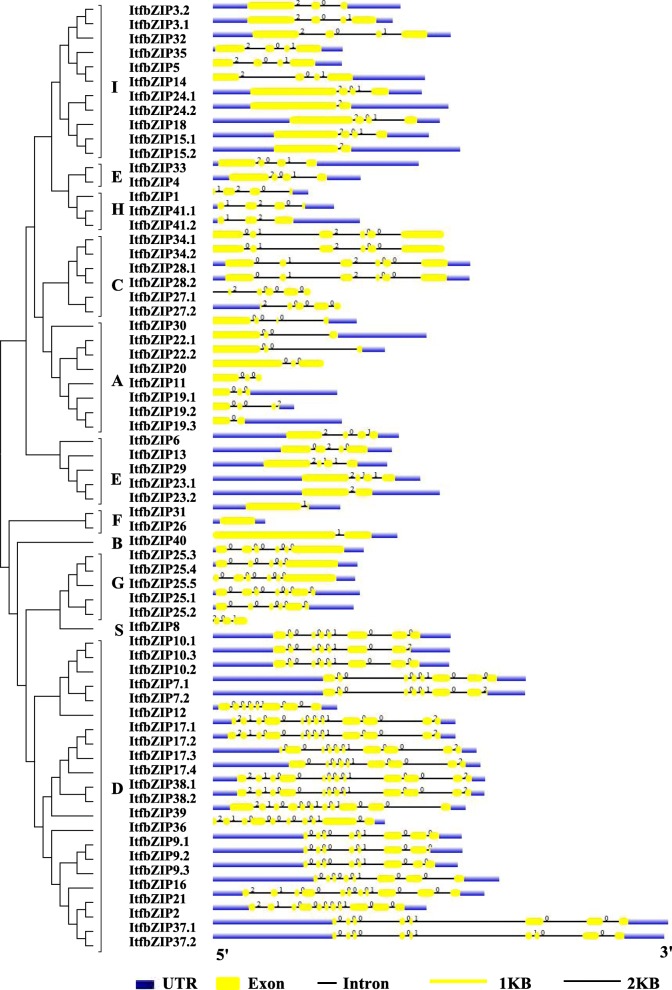
Exon-intron structures of *ItfbZIP* genes. The phylogenetic tree on the left was constructed using MEGA 7 software based on the full length sequences of the ItfbZIP proteins.Yellow rectangles represent exons, blue rectangles represent the untranslated regions, black thin lines represent introns

The intron/exon structure was determined by analysing genomic DNA with full-length *ItfbZIP* CDS sequences (Fig. 4). Results indicated that the *ItfbZIPs* contains at least one intron except *ItfbZIP26* with no intron. The D group *ItfbZIPs* contain 7 to 11 introns, followed by the G group with 4 to 6 introns, the C group with 4 to 5 introns, the H group with 2 or 3 introns, the S group with 3 introns, the A, E and I group with 1 to 3 introns, the B group with 1 intron, and finally the F group with 0 or 1 intron. The different *ItfbZIP* transcripts encoded by same *ItfbZIP* gene have similar exon/intron structure and intron phase patterns.

Conserved domain analysis of the ItfbZIP proteins was performed using the SMART and Pfam databases. The DNA binding ability and the heterozygosity of bZIP TFs are determined by the bZIP conserved domain, which mainly includes the basic domain (N-× 7-R/K) and the leucine zipper domain (L-× 6 -L-× 6-L). The results indicate that the majority of the ItfbZIP family members contain a highly conserved bZIP domain visualized by genedoc software in Additional file 4: Figure S1. In the basic region, only the Asp of five ItfbZIP (ItfbZIP-6, − 13, − 23.1, − 23.2, − 29) in the E subfamily is replaced by Lys/Gln, and the Arg/Lys of five ItfbZIP (ItfbZIP-25.1, − 25.2, − 25.3, − 25.4, − 25.5) in the G subfamily are replaced by Ile. Interestingly, the conventional 9 amino acid residues are replaced by 12 amino acid residues at R/K-× 9-L region of ItfbZIP11, which is similar to OsbZIP34 in rice [3]. The leucine zipper region contains a Leu at 7th amino acid position (9 amino acids after R/K in the C-terminal extension), but sometimes Leu is replaced by other hydrophobic residues (IIe, Val and Met), such as ItfbZIP12, ItfbZIP13, ItfbZIP23.1, ItfbZIP23.2, ItfbZIP27.1, ItfbZIP27.2.

### C*is*-element prediction of *ItfbZIP* genes

To understand the potential regulatory mechanisms of *ItfbZIP* gene responding to abiotic stress, *cis*-elements in the *ItfbZIP* promoter were analyzed using Analysis Navigator (PlantPAN 2.0) database and plantCARE (Additional file 5: Table S4). Every promoter of *ItfbZIP* gene has at least one stress related *cis*-element. Among these elements, 96% of the *ItfbZIP* genes contained multiple stress-responsive regulatory elements (HSE stress response element, MBS response element for drought stress, low-temperature stress response element LTR and phosphate starvation responsive element P1BS). This finding indicates that the expression of these *ItfbZIP* genes is regulated by different abiotic stress. In addition, these *ItfbZIP* promoters contain hormone response elements (Abscisic acid response element ABRE, gibberellin response element P-box, GARE-motif, TATC-box, auxin response element TGA-box, ethylene response element ERE, MeJA response element TGCCG-Motif and CGTCA-motif, and salicylic acid responsive element TCA-element). This finding suggests that *ItfbZIPs* participate in the regulation of plant growth and development and abiotic stress adaption. This result is also consistent with previous *cis*-acting elemental analyses of the bZIP TFs in *Hordeum vulgare* L, six *fragaria* species, *Vigna radiata*, *Vigna angularis* and *Solanum tuberosum* [46–49].

### Interaction network of the ItfbZIP proteins

**Fig. 5:**
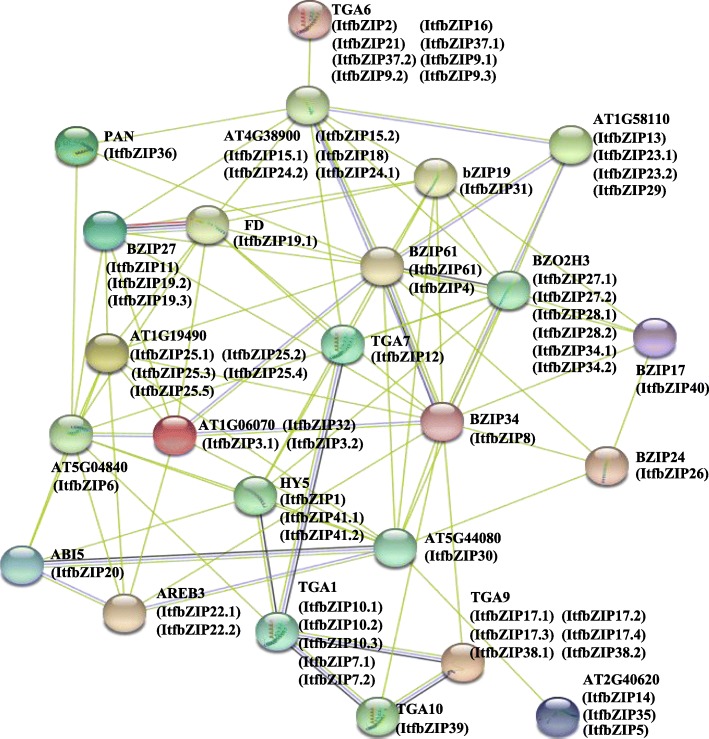
Functional interaction networks of ItfbZIP proteins in *Ipomoea trifida* according to orthologues in *Arabidopsis thaliana*. Network nodes represent proteins, and edges represent protein-protein associations

Understanding the functional relationships of the ItfbZIP proteins is important for studying the family’s regulatory pathways. Thus, we used the STRING software to construct an *ItfbZIP* gene interaction network based on *Arabidopsis thaliana* orthologous genes, systematically analysing the interactions of ItfbZIP proteins (Fig. 5). Among the proteins, ABI5 (ItfbZIP20), AREB3 (ItfbZIP-22.1, − 22.1), AT5G44080 (ItfbZIP6), PAN (ItfbZIP36), TGA1 (ItfbZIP-7.2, − 7.1, − 10.1, − 10.2, − 10.3), TGA10 (ItfbZIP39), TGA6 (ItfbZIP-2, − 16, − 21, − 37.1, − 37.2, − 9.1, − 9.2, − 9.3), TGA7 (ItfbZIP12) and TGA9 (ItfbZIP-17.1, − 17.2, − 17.3, − 17.4, − 38.1, − 38.2) are involved in the KEGG signalling pathway of plant hormone signal transduction (ID 4075). TFs that serve other functions were also observed. HY5 (ItfbZIP-1, − 41.1 and − 41.2) is involved in the downstream reaction of cryptochrome (CRY1 and CRY2) signals. bZIP19 (ItfbZIP31) regulates the expression of zinc transporters ZIP3, ZIP4, ZIP5 and ZIP9 during root growth. BZIP24 and BZIP17 (ItfbZIP40) are involved in salt and osmotic pressure responses. BZIP34 (ItfbZIP8) may play an important role in controlling metabolic pathways that regulate cell transport, and lipid metabolism. BZIP27 (ItfbZIP-11, − 19.2 and − 19.3) promotes TFs required for transition to flowering. Therefore, the study of the interactions of the ItfbZIP family members provided us new research ideas for further exploring of new functions of ItfbZIPs.

### Homology modeling of ItfbZIP proteins

**Fig. 6:**
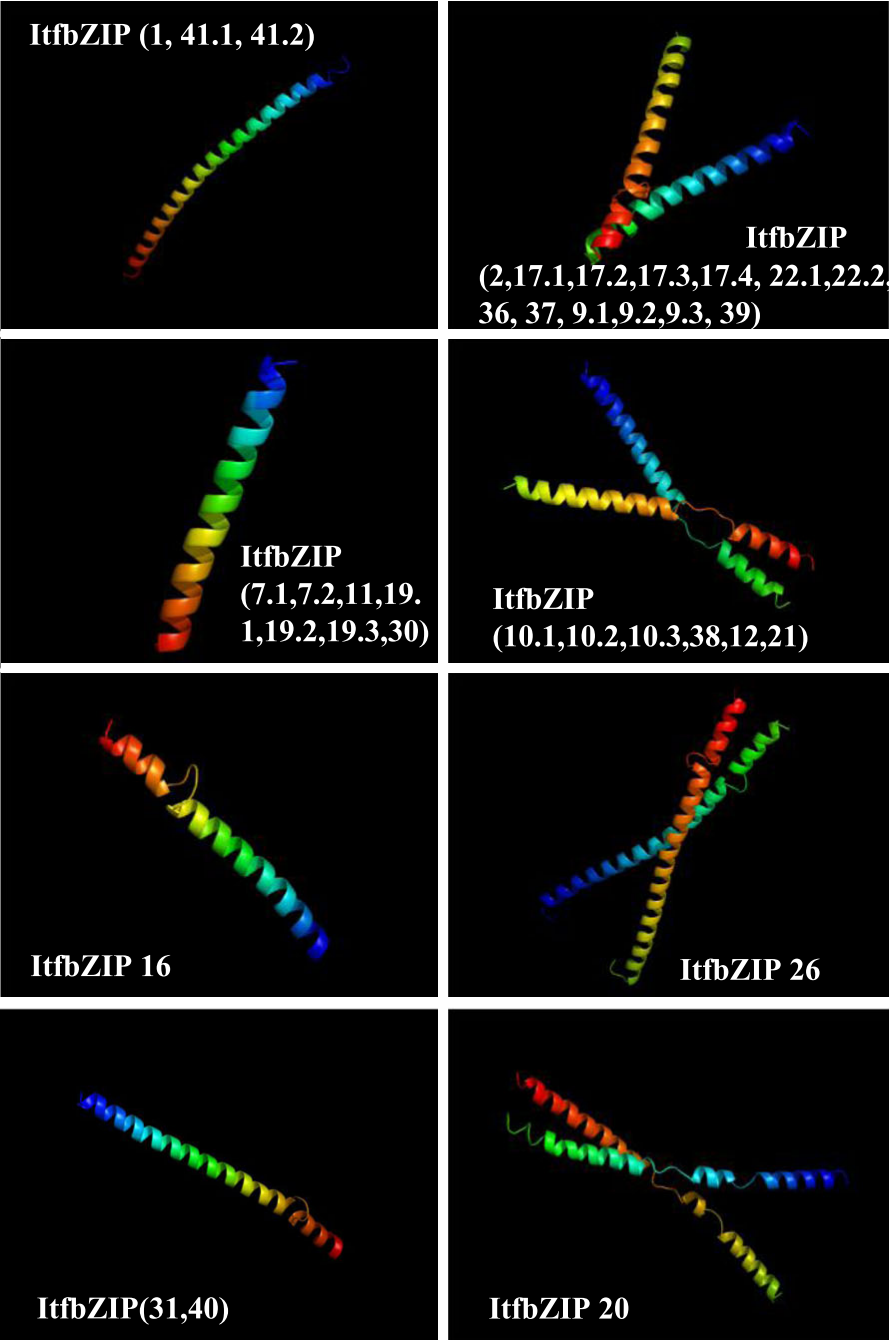
Homology modeling of ItfbZIP proteins. The figure shows the three-dimensional structural models of 34 ItfbZIP proteins (distributed in the A (ItfbZIP-11, − 19.1, − 19.2, − 19.3,-20, − 22.1, − 22.2, − 30), B (ItfbZIP40), D (ItfbZIP-2, − 7.1, − 7.2, − 10.1, − 10.2, − 10.3, − 12, − 9.1, − 9.2, − 9.3, − 16, − 17.1, − 17.2, − 17.3, − 17.4, − 21, − 36, − 37, − 38, − 39), F (ItfbZIP-26, − 31) and H (ItfbZIP-1, − 41.1, − 41.2) subfamilies) predicted by Swiss-Model software

Swiss-Model was used to analyze three-dimensional structural homology modeling of ItfbZIP amino acid sequences. Because SWISS-model does not predict effectively for sequences with low homology, we retained 34 models of ItfbZIP proteins with homology higher than 30%. Figure 6 shows the ItfbZIP three-dimensional structural models in the subfamilies A (ItfbZIP-11, − 19.1,-19.2, − 19.3, − 20, − 22.1, − 22.2, − 30), B (ItfbZIP40), D (ItfbZIP-2, − 7.1, − 7.2, − 10.1, − 10.2, − 10.3, − 12, − 9.1, − 9.2, − 9.3, − 16, − 17.1, − 17.2, − 17.3, − 17.4, − 21, − 36, − 37, − 38, − 39), F (ItfbZIP-26, − 31) and H (ItfbZIP-1, − 41.1, − 41.2). The results show that all proteins have α-helices but no β-sheets. The ItfbZIPs in H subfamily mainly contain an α-helices. The A, B, D and F subfamily ItfbZIPs contain a few random coiled structures besides α-helices. At the same time, the number of α-helices and random coils among different members in the same subfamily are different, such as A, D and F subfamily, suggesting that different members in the same subfamily may have different functions.

### Gene expression analysis of *ItfbZIPs* in various tissues of *Ipomoea trifida*

**Fig. 7:**
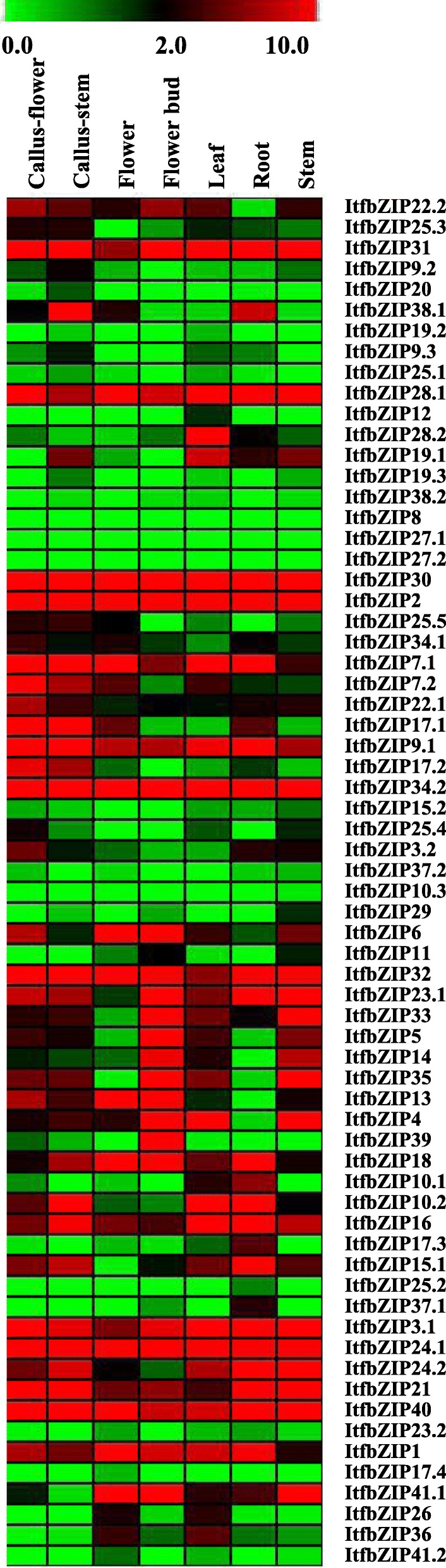
Relative expression levels of *ItfbZIP* genes across various tissues. A heat map with clustering is created based on the FPKM value of ItfbZIPs. The coloured scale varies from green to red, indicating relatively low or high expression

**Fig. 8:**
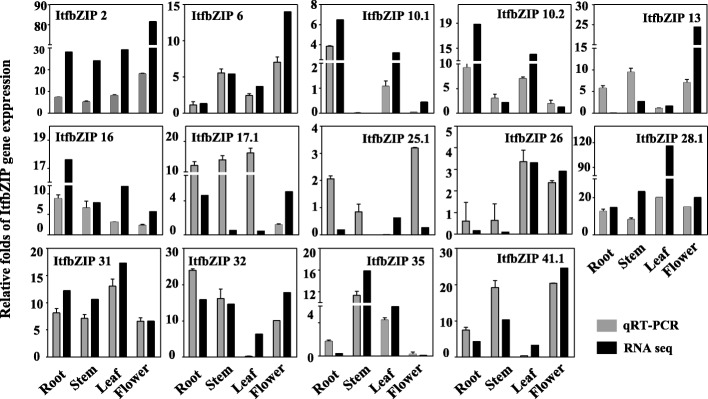
The comparison between quantitative RT-PCR data and RNA-seq data. The relative expression of the 14 selected *ItfbZIP* genes was analyzed by qRT-PCR. The GAPDH transcript levels were used for normalization. The y-axis represents the relative expression of the fold. Characters on the x-axis represent various tissues, Error bars indicate standard deviation. qRT-PCR data represented by gray bars, and black bars represent RNA-seq data

To gain insights into the role of the *ItfbZIP* genes in the growth and development, we used RNA-seq data from seven tissues (callus_flower, callus_stem, flower, flower bud, leaf, root and stem) to study the expression patterns of the *ItfbZIP* genes in different tissues. As shown in Figs. 7, 40% of ItfbZIP transcripts share similar expression patterns in the roots, stems, leaves, flower buds and flowers. For example, *ItfbZIP2* and *ItfbZIP30* were highly expressed in seven tissues, whereas *ItfbZIP10.3* and *ItfbZIP38.2* were lowly expressed in these tissues. Moreover, the expression patterns of different transcripts of the same gene differ in each tissue. For example, *ItfbZIP9.1* was highly expressed in each tissue, whereas *ItfbZIP9.2* and *ItfbZIP9.3* were lowly expressed. Interestingly, *ItfbZIP28.2* was only highly expressed in the leaf. *ItfbZIP4* was lowly expressed only in the roots. *ItfbZIP17.2* was highly expressed only in the callus_flower and callus_stem. To confirm the expression profiles of *ItfbZIP* gene obtained from RNA-seq analysis, we randomly selected 14 ItfbZIP genes to investigate their expression in four different tissues (stem, root, leaf and flower) by qRT-PCR (Fig. 8). The results show that the qRT-PCR results matched well with RNA-seq data, such as ItfbZIP2, ItfbZIP6, ItfbZIP10.1, ItfbZIP10.2, ItfbZIP16, ItfbZIP26, ItfbZIP28.1, ItfbZIP31, ItfbZIP32, ItfbZIP41.1.

### Expression of *ItfbZIP* genes under abiotic stress and ABA treatment

**Fig. 9:**
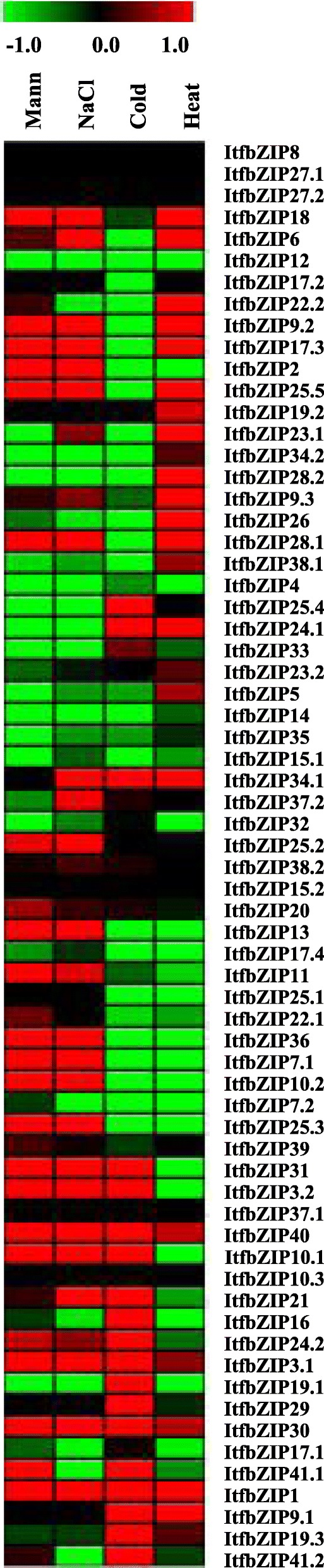
Expression pattern of *ItfbZIP* genes under abiotic stress. A heat map created with clusters based on the ItfbZIP FPKM value. Red color indicates a high expression of the relevant gene

**Fig. 10:**
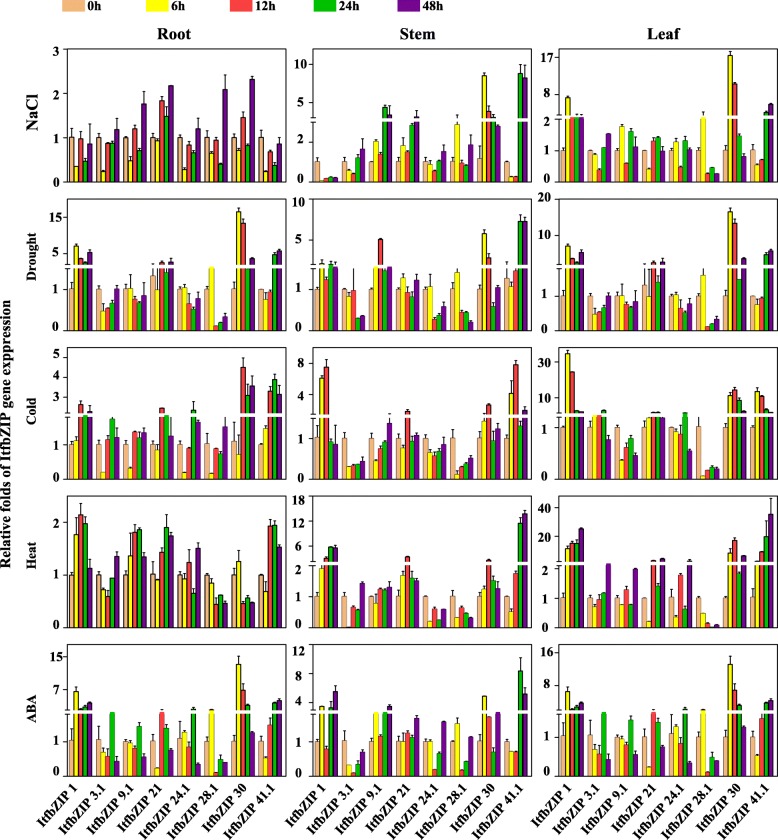
Gene expression confirmed by qRT-PCR under salt, drought, cold and heat stresses and ABA treatment. The expression of 0 h was set up as 1 fold. The y-axis indicates the fold changes of relative gene expression comparing with 0 h the expression. Characters on the x-axis represent selected 8 *ItfbZIP* genes. Error bars indicate standard deviation. The expression levels at 0 h, 6 h,12 h,24 h and 48 h are indicated by powder, yellow, red, green, and purple bars, respectively

The bZIP TF family plays an important role in plant stress response. Thus, it is very necessarily to investigate the expression of *ItfbZIPs* under abiotic stress and hormonal treatment. Figure 9 shows the expression of *ItfbZIP* genes under drought, salt, cold and heat stress. Among them, the expression of *ItfbZIP1*, *ItfbZIP3.1* and *ItfbZIP40* were all up-regulated under these four abiotic stresses. The expression of *ItfbZIP10.1*, *1tfbZIP3.2*, *1tfbZIP21* and *ItfbZIP31* were up-regulated under drought, salt and cold stress. Six genes (*ItfbZIP -25.5*, *− 28.1*, *− 18*, *− 6*, *− 17.3* and *− 9.2*) were up-regulated under heat, salt and drought stress. Only *ItfbZIP34.1* was up-regulated under salt, heat and cold stress. The expression of eight *ItfbZIP* genes (*ItfbZIP-25.3*, *− 25.2*, *− 7.1*, *− 2*, *− 13*, *− 36*, *− 10.2*, and *− 11*) were up-regulated under salt and drought stress. Seven *ItfbZIP genes (ItfbZIP-4*, *− 12*, *− 14*, *− 15.1*, *− 7.2*, *− 17.4*, *− 35)* were all down-regulated under drought, cold, salt and heat stress. To verify the RNA-seq data and further clarify the expression pattern of the *ItfbZIPs* in detail, gene expression was analyzed by qRT-PCR in plants root, stem and leaf under salt, drought, cold, heat and ABA treatment. Eight *ItfbZIP* genes (*ItfbZIP-1*, *− 3.1*, *− 9.1*, *− 21*, *− 24.1*, *− 28.1*, *− 30*, *− 41.1*) which were up-regulated under at least under one of the four stresses are selected for qRT-PCR analysis (Fig. 10). The results show that the *ItfbZIP* genes respond to salt, drought, cold, heat or ABA treatment differently. In roots, the gene expression of *ItfbZIP1* and *ItfbZIP41.1* were up-regulated under drought, cold, heat and ABA stress treatments. And the gene expression of *ItfbZIP21* was up-regulated at 12 h and 24 h under salt, drought, cold, heat and ABA stresses. In the stems, *ItfbZIP41.1* was up-regulated at 24 h and 48 h under these 4 stresses and ABA treatment. In the leaves, the expression of *ItfbZIP1* and *ItfbZIP30* were up-regulated under drought, cold, heat and ABA stresses. Under ABA treatment, the expression of eight *ItfbZIP* genes were induced at least in one tissue (root, stem, leaf) and at one time point (6 h, 12 h, 24 h or 48 h). The expression of ItfbZIP3.1 was down-regulated at every time point in stem after ABA.

## Discussion

Sweet potato is an important grain, health care and industrial raw material crop that features wide adaptability, high yield and strong resistance to environmental stress. China has the largest planting area and highest yield of sweet potato in the world. However, the genetic background of cultivated sweet potato is complex. The cytogenetics and genomics basis of sweet potato are much weaker than that of other food crops (such as rice, corn and wheat) [50]. However, the genome of *Ipomoea trifida* as most probably the progenitor of the sweet potato was released recently, which is very helpful for the study of the sweet potato genetic improvement. BZIP is the most widely distributed TF involved in stress resistance in the eukaryote community. In plants, it plays an important role not only in growth and development but also in the response to abiotic and biotic stresses. To date, most of the studies on the physiological and molecular mechanisms of *bZIP* are focused on *Arabidopsis thaliana* and rice plants but not on *Ipomoea trifida*. Whole genome analysis *ItfbZIP* genes are usually used to screen new varieties of plants with high yield and resistance to stress condition. Genome-wild study of *ItfbZIPs* in *Ipomoea trifida* has an important guiding role in the further in-depth study of *bZIP* gene function and molecular breeding of sweet potato.

### Evolution of *ItfbZIP* genes

In this study, 41 *ItfbZIP* genes encoding 66 transcripts were identified. The number of *ItfbZIP* genes is significantly less than that of other Solanales and cereal crops that have been genome sequenced (Fig. 2), indicating that the *ItfbZIP* gene family shrinks during the long evolutionary process. All the ItfbZIPs were predicted to be localized in the nucleus; this finding is consistent with the reported experiment results that the CAbZIP1 of pepper, the OsABI5 of rice and the TabZIP1 of wheat are all localized in the nucleus [51–53]. The number of *ItfbZIP* genes in every chromosome of *Ipomoea trifida* is in the range of 1 to 10 (Fig. 1). And *ItfbZIPs* in *Ipomoea trifida* showed the similar chromosomal disproportionate distribution pattern with *bZIP* genes in *Hordeum vulgare* [49], *Cucumis sativus* [11] and *Medicago truncatula* [54]. Segmental and tandem duplication analysis indicates that the contribution of tandem duplication was limited for *ItfbZIPs* and segmental duplication played a dominant role in the amplification of *ItfbZIP* gene, which was consistent with the findings in *Arabidopsis thaliana*, rice and grape [10, 28, 32]. Based on the phylogenetic relationship, *ItfbZIP* family are divided into ten subfamilies, namely, A, B, C, D, E, F, G, H, I and S (Fig. 3), which is similar to bZIP subfamily classification reported in *Maninot esculenta*, *Musa acuminata* and *Vitis vinifera*. [10, 12, 55]. Moreover, the analysis results of intron/exon gene structure also support phylogenetic analysis. That is, the regularity of the number and distribution of introns is consistent with the subfamily classification from evolutionary tree (Fig. 4). The DNA binding ability and heterogeneity of the bZIP transcription factor are determined by the bZIP domain. Asp (− 18), Arg (− 10) and Leu (+ 1, + 8, + 15) are key and invariant sites in the bZIP domain (N(− 18)-× 7-R/K(− 10)-× 9-L(+ 1)-× 6 -L(+ 8)-× 6-L(+ 15)) [9, 56]. Most of the ItfbZIPs have highly conserved bZIP domains, while a few ItfbZIPs show some substitutions, such as ItfbZIP-6, − 11 -13, − 23.1, − 23.2, − 29, -25.1, − 25.2, − 25.3, − 25.4, − 25.5 (Fig.S1). Protein homology modeling indicated that α-helix is the main structure of ItfbZIP proteins, which is supported by previous research results: the leucine zipper in the conserved region of bZIP forms one amphiphilic α-helices, which is the dimerization of bZIP protein before binding to DNA [57].

### Expression and potential functions of *ItfbZIP* genes

bZIP TFs play essential roles in many biological processes, such as plant growth, development and stress responses et al. In *Arabidopsis thaliana*, AtbZIP16 can regulate early seedling development by integrating light and hormone signalling pathways and promote seed germination and hypocotyl elongation in the early stage of seedling germination [58]. AtbZIP28 binds to the endoplasmic reticulum membrane and plays an important role in resistance to heat stress [59]. AtbZIP10-LSD1 regulates basic defence and cell death in *Arabidopsis thaliana* after infection [60]. In rice, OsbZIP12 is a positive regulator of ABA signalling, conferring ABA-dependent drought tolerance [61]. And OsbZIP23 and OsbZIP72 also increase drought resistance through the ABA pathway [29, 62]. Overexpression of *OsbZIP66* and *OsbZIP71* enhances the drought tolerance in rice plants [63, 64]. And overexpression of the *OsbZIP60* enhances heat and drought resistance [65]. Some *bZIP* genes have also been studied in Solanales plants. For example, over-expression of *CaHB1* gene in tomato can enhance tomato salt tolerance [66]. Expressing tomato *SIAREB* in *Arabidopsis thaliana* triggers AtRd29A, AtCOR47 and SICI7-like dehydrin in drought and salt responses [67]. Overexpressing *SlAREB1* in tomato can increase the plant tolerance to salt and water stresses [68]. Tobacco bZIP transcription factors TGA2.2 and TGA2.1 have distinct roles in plant defense and plant development [69]. In our study, ItfbZIPs which have high homology with *Arabidopsis thaliana* and *Solanum lycopersicum* bZIPs may play similar roles in specific biological processes.

Protein phosphorylation is one of the most important post-translational modifications and plays an essential role in regulating the activity of TFs. Regulating the TF’s entry into the nucleus may also change the DNA-binding ability or activity of the TF. The activation of rice TREB protein and *Arabidopsis thaliana* ABI5 protein require phosphorylation [70, 71]. Activation of threonine/serine casein kinase II (CKII) phosphorylation of G-box-binding factor 1 (GBF1) in *Arabidopsis thaliana* is associated with plant senescence [72]. bZIP transcription factors AREB1, AREB2 and ABF3 are phosphorylated by SNF1-related protein kinase SRK2D, which activates the cascade response of plants to drought and water stress depending on ABA signaling pathway [36]. The predicted phosphorylation sites of ItfbZIP proteins are shown in Table 1. All ItfbZIP proteins have 4 to 18 phosphorylation sites, suggesting that ItfbZIPs may act through post-transcriptional phosphorylation modification.

*bZIP* gene is involved in plant tissue and organ development. Investigation of tissue-specific gene expression pattern helps us get some hints about the gene function. RNA-seq data were used to analyse the expression pattern of *bZIP* genes in the roots, stems, leaves and flowers of *Ipomoea trifida.* As shown in Figs. 7, 40% of the ItfbZIP transcripts have similar expression patterns, and 12 genes are highly expressed in these four tissues. These findings indicate that these genes may play an important role in the plant growth development. Interestingly, *ItfbZIP28.2* is highly expressed only in the leaf, *ItfbZIP4* is lowly expressed only in the roots and *ItfbZIP17.2* is highly expressed only in the callus flower and callus stem, indicating that these genes function in specific organs during the growth and development of *Ipomoea trifida*.

To date, increasing evidence shows that *bZIP* genes are involved in hormonal/abiotic stress and related signal transduction pathways. In *A. thaliana*, TGA2, TGA5 and TGA6 are essential activators of defence responses induced by salicylic acid (SA)/ethylene [73]. TGA7 is involved in plant drought stress. TGA9 and TGA10 are involved in plant immune responses [74]. Combination analysis of the *cis*-acting elements and protein interaction networks suggests that *ItfbZIPs* protein may participate in KEGG signalling pathway and plant hormone signal transduction (ID 4075) (Fig. 5 and Additional file 5: Table S4). In addition, PAN (ItfbZIP36), TGA1 (ItfbZIP-7.2, − 7.1, − 10.1, − 10.2 and − 10.3), TGA10 (ItfbZIP39), TGA6 (ItfbZIP-2, − 16, − 21, − 37.1, − 37.2, − 9.1, − 9.2 and − 9.3), TGA7 (ItfbZIP12), and TGA9 (ItfbZIP-17.1, − 17.2, − 17.3, − 17.4, − 38.1 and − 38.2) are all Group D ItfbZIPs TFs. And D subfamily genes contain *cis*-acting elements associated with biotic and abiotic stresses. The gene expression of *ItfbZIP7.1* and *ItfbZIP36* was up-regulated under drought and salt stresses and down-regulated under cold and heat stresses. The expression of *ItfbZIP10.1* was up-regulated under three stresses (droguht, salt and cold) but down-regulated under heat stress. *ItfbZIP 17.3* was down-regulated under cold stress and up-regulated under the other three stresses (droguht, salt and heat). This finding suggests Group D *ItfbZIPs* have specific meaning for abiotic stress response and related signal transduction. The different gene expression patterns of *ItfbZIPs* indicate *ItfbZIPs* play different roles under various abiotic stress conditions (Fig. 9 and Fig. 10).

## Conclusion

*Ipomoea trifida* genome has 41 Itf*bZIP* genes. These genes are localized on 14 chromosomes and are uniformly named according to their chromosomal location. According to the phylogenetic analysis of *Arabidopsis thaliana* and *Solanum lycopersicum*, *ItfbZIP* gene family is divided into ten subfamilies which have certain genetic relationships and have similar biological functions. The phylogenetic tree can be used to infer the diversity and conservation of genes during evolution. This finding is supported by intron/exon structures, *cis*-acting elements and protein interaction networks. The expression patterns of the *bZIP* gene family members are also analyzed using RNA-Seq data and qRT-RNA. Results reveal that bZIP TFs play an important role in plants abiotic stress responses. This study provides a theoretical basis for the application of *ItfbZIP* genes in molecular breeding of sweet potato.

## Methods

### Plant materials and stress treatments

*Ipomoea trifida* plants were collected from the Sweet Potato Research Institute, Xuzhou Academy of Agricultural Sciences, National Sweet Potato Industry System, China. The plants were grown in soil and vermiculite (1:1) under the greenhouse conditions of 28/22°Cday/night and a photoperiod of 16 h light/8 h dark. And *Ipomoea trifida* (2x) were watered every 5 days. In order to analyze different tissues expression profiles of *ItfbZIP* genes, roots, stems and leaves were sampled from 4-week-old *Ipomoea trifida* plants and flowers were sampled from 2-month-old *Ipomoea trifida* plants. To study the expression patterns related various stress and hormone treatments, 4 week old *Ipomoea trifida* plants were subjected to 200 mM NaCl, 300 mM mannitol, 50 uM abscisic acid solution, respectively. For cold and heat treatment, 4 week old *Ipomoea trifida* plants were subjected to 12 °C and 40 °C, respectively. Roots, stems and leaves of the above treated plants were sampled after 0 h, 6 h, 12 h, 24 h and 48 h treatments.

### Acquisition and identification of *bZIP* genes in *Ipomoea trifida*

The genome annotations of *Ipomoea trifida*, *Arabidopsis thaliana* and *Solanum lycopersicum* were downloaded from Sweetpotato Genomics Resource (http://sweetpotato.plantbiology.msu.edu/), TAIR (https://www.arabidopsis.org/. jsp) and the Sol Genomics Network (https://solgenomics.net/). Firstly, the candidate bZIP TF members were identified using the Pfam database (http://pfam.xfam.org/search#tabview=tab1) and HMMER 3.0 software (http://hmmer.janelia.org/). Then, the candidate *ItfbZIP* members were further confirmed if they contained the bZIP domain via using online programmes NCBI-CDD (https://www.ncbi.nlm.nih.gov/cdd/ Structure/cdd/wrpsb.cgi) and SMART database (http://smart.embl.de/).

### Chromosomal distribution and synteny analysis of *ItfbZIPs*

The *ItfbZIPs* gene were mapped to the *Ipomoea trifida* chromosome based on the chromosomal location provided in the Sweetpotato Genomics Resource (http://sweetpotato.plantbiology.msu.edu/). Gene duplication events were analyzed using the Multiple Collinearity Scan toolkit (MCScanX) using default parameters [75]. Finally, the visualization was generated by the circos (version 0.69) (http://circos.ca/) [76].

### Protein properties, gene structure and promoter region prediction of *ItfbZIP* genes

The molecular weight (MW) and the theoretical isoelectric point (pI) of the ItfbZIP proteins were calculated using the online tool ExPASy (http://e xpasy.org/tools/). The subcellular location of these genes was passed through the software WoLF PSORT (https://wolfpsort.hgc.jp/) forecast. Phosphorylation analysis of the *ItfbZIP* genes was conducted by the online tool P3DB (http://www.p3db.org/) [77]. The *ItfbZIP* genes were graphically displayed on the *Ipomoea trifida* chromosome. The *bZIP* gene intron-exon structure map was obtained using the *ItfbZIP* gene coding sequences and the corresponding genomic sequences together into the GSDS v2.0 (http://gsds.cbi.pku.edu.cn/) [78]. The Plantpan2.0 (http://plantpan2.itps.ncku.edu.tw/) and plantCARE (http://bioinformatics.psb.ugent.be/webtools/plantcare/html/) were used to detect *cis*-elements in the approximately 2000 bp promoter region of each *ItfbZIP* gene [79].

### Phylogenetic tree construction, domain identification and protein homology modeling

The ClustalX program was used to perform multiple sequence alignments of the bZIPs of *Ipomoea trifida*, *A. thaliana* and *S. lycopersicum.* The Maximum Likelihood method was used to construct the phylogenetic tree by MEGA7 programme [80, 81]. The Bootstrap value was set to 1000. The sequence alignment of the conserved domain (bZIP domain) of ItfbZIPs was performed by using the online SMART (http://smart.embl-heidelberg.de/) and Pfam database (http://pfam.xfam.org/search# tabview = tab1), and then visualized using genedoc. STRING software was used to construct functionally interacting protein networks with a confidence parameter set at 0.15 threshold. Online SWISS-MOLD (https://www.swissmodel.expasy.org/) [82] and Pymol software were used for homology modeling of ItfbZIP proteins.

### Transcriptome analysis

The *ItfbZIP* RNA-seq data were downloaded from the Sweetpotato Genomics Resource (http://sweetpotato.plantbiology.msu.edu/). The data are shown in Additional file 6: Tables S5 and Additional file 7: Table S6. The *ItfbZIP* gene expression levels were calculated as fragments per kilobase of exon per million fragments mapped (FPKM). Heat maps of gene expression profiles were drawn using MeV 4.9.0 software.

### Quantitative real-time PCR analysis

RNA prep Pure Kit (Tiangen Biotech, Beijing, China) was used to extract total RNA. First-strand cDNA was synthesized using the PrimescriptTM RT reagent kit (Tsingke, Nanjing, China). And then the reverse transcription product was diluted and used as qRT-PCR template. Glyceraldehyde-3-phosphate Dehydrogenase (GAPDH) gene of *Ipomoea trifida* was used as internal control to evaluate relative gene expression levels. Primers were designed using online Primer 3 (http://bioinfo.ut.ee/primer3-0.4.0/), and all the primers were shown in Additional file 8: Table S7. The experiments were conducted for 3 repetitions and the data were calculated using the 2^-△△Ct^ method [83].

## Additional files

Additional file 1: **Table S1.** Chromosomal locations and segmental duplication of *ItfbZIP* genes. (EMF 15058 kb)

Additional file 2: **Table S2.** Distribution of bZIP transcription factors in eukaryotes. (XLSX 11 kb)

Additional file 3: **Table S3.** Accession numbers of *bZIP* genes in *Ipomoea trifida*, *Arabidopsis thaliana* and *Solanum lycopersicum. (XLSX 10 kb)*

Additional file 4: **Figure S1.** The sequence alignment of the conserved domain (bZIP domain) in ItbZIP transcription factors. The primary structure of the bZIP domain and highly conserved residues of the bZIP domain consensus sequence [N-X7-R/K-X9-L-X6-L-X6-L] are shown at the top of the picture. Vertical bars with different colors on the left show different subfamilies. The black and gray shades in the picture present the same and similar amino acids, respectively. (XLSX 23 kb)

Additional file 5: **Table S4.** Cis-elements associated with abiotic stress in promoter region of *ItfbZIPs. (XLS 38 kb)*

Additional file 6: **Table S5.** Relative expression levels of *ItfbZIPs* in various tissues. (XLSX 30 kb)

Additional file 7: **Table S6.** Expression pattern of *ItfbZIP*s under abiotic stresses. (XLSX 38 kb)

Additional file 8: **Table S7.** Primers of the *ItfbZIP* genes and housekeeping gene for qRT-PCR. (XLSX 10 kb)

## Abbreviations

AA: Amino acid; pI, Isoelectric points
ABA: Abscisic acid
bZIP: Basic leucine zipper
ItfbZIP: Basic leucine zipper of *Ipomoea trifida*
KEGG: Kyoto Encyclopedia of Genes and Genomes
Leu: Leucine
MW: Molecular weights
TFs: Transcription factors
